# The diversity of social complexity in termites

**DOI:** 10.1098/rspb.2023.2791

**Published:** 2024-06-05

**Authors:** Lewis Revely, Paul Eggleton, Rebecca Clement, Chuanyu Zhou, Tom R. Bishop

**Affiliations:** ^1^ Soil Biodiversity Group, Department of Life Sciences, The Natural History Museum, London SW7 5BD, UK; ^2^ Centre for Biodiversity and Environmental Research, Department of Genetics, Evolution and Environment, University College London, London WC1E 6BT, UK; ^3^ Computational Biology Institute, George Washington University, Washington, DC 20052, USA; ^4^ School of Biosciences, Cardiff University, Cardiff CF10 3AX, UK; ^5^ Department of Zoology and Entomology, University of Pretoria, Pretoria, South Africa

**Keywords:** major evolutionary transitions, social evolution, termites, individuality, museum collections

## Abstract

Sociality underpins major evolutionary transitions and significantly influences the structure and function of complex ecosystems. Social insects, seen as the pinnacle of sociality, have traits like obligate sterility that are considered ‘master traits’, used as single phenotypic measures of this complexity. However, evidence is mounting that completely aligning both phenotypic and evolutionary social complexity, and having obligate sterility central to both, is erroneous. We hypothesize that obligate and functional sterility are insufficient in explaining the diversity of phenotypic social complexity in social insects. To test this, we explore the relative importance of these sterility traits in an understudied but diverse taxon: the termites. We compile the largest termite social complexity dataset to date, using specimen and literature data. We find that although functional and obligate sterility explain a significant proportion of variance, neither trait is an adequate singular proxy for the phenotypic social complexity of termites. Further, we show both traits have only a weak association with the other social complexity traits within termites. These findings have ramifications for our general comprehension of the frameworks of phenotypic and evolutionary social complexity, and their relationship with sterility.

## 1. Introduction

Life comes in a bewildering diversity of forms. One of the most striking and essential features about living organisms is their astonishing array of social interactions. Chromosomes cooperate with other chromosomes within cells; cells associate with other cells to form multicellular organisms; and individual insects live with other, related insects to form cohesive colonies [1–5]. The variety and depth of these cooperations can be thought of as social complexity. Social complexity, which encompasses the variety and depth of cooperation in such systems, forms a framework to understand phenotypic evolution and the ecology of communities [1–5].

There are several ways to describe, compare and understand variation in social complexity. Here, we focus on two: evolutionary and phenotypic views of social complexity. The evolutionary viewpoint (box 1) asks why social complexity exists at all and investigates the factors determining why individual units cooperate. Under an evolutionary social complexity framework, the diversity of life can be explained by a stepwise progression whereby solitary individuals (e.g. bacteria or mason bees) exist on their own, but can form social groups (e.g. slime moulds or honeybees). In turn, social groups can come together and form interdependent fitness-maximizing individuals (all units working together as a whole). These shifts from one level of sociality to another are termed major evolutionary transitions (e.g. as seen in red algae or mound-building termites) [1]. Crucially, this stepwise progression can be recursive, creating a nested hierarchy of cooperating units, all working as part of the highest-level individual (box 1) [3]. For instance, some social insects are highly integrated societies composed of many individuals, which are themselves made up of cooperating cells, which in are in turn collections of cooperating genes. Therefore, we use the term ‘evolutionary social complexity’ to describe the degree to which a group has transitioned to a fitness maximizing individual at a given level. These transition stages provide us with a valuable tool for understanding the intricacies of social behaviour and how it evolves.

Box 1. **Glossary**

**Conflict**. This would refer to evolutionary conflict of interest between members of a social group over actions like reproduction. This can be potential conflict when there are differing inclusive fitness optima, such as having higher relatedness of workers to their own offspring compared with their siblings. This may not become actual conflict however (for instance, when there is worker reproduction within a colony while the queen is present).

**Fertile workers**. workers that can become dispersing primary reproductives that form their own colony or replacement reproductives able to take over a nest if a parent dies.

**Foraging termites** (also referred to as separate-piece or multiple piece nesters). Species that live in a well-defined nest where workers, at some point in the colony cycle, will leave the nest to forage. This means the colony longevity is not limited to the availability of food. All foraging termites have true workers which can be thought to have reduced potential and therefore are at least functionally sterile. These are found in the Mastotermitidae, Hodotermitidae, most Rhinotermititdae and all Termitidae.

**Functional sterility**. When the working unit in a group can become a reproductive only under extreme circumstances. For instance, in termites the apterous (working/somatic) and nymphal (reproductive/germline) lines have incomplete separation where workers can still become reproductives if for example a parent dies but are unable to become dispersing reproductives. Some species with functional sterility, like *Mastotermes* and *Reticulitermes*, have worker-derived reproductives in the nest while the primary reproductive is still present but the ability of a worker to become a worker is still much reduced compared with species with fertile workers.

**Individual**. A collective that adheres to the conditions needed for a major evolutionary transition (i.e. a group whose lower units are interdependent and have aligned interests).

**Major evolutionary transition**. A change in the way that heritable information is stored and transmitted, concentrating on transitions that lead to a new form of individual. This requires two conditions to be met: (1) entities capable of independent replication before the transition can replicate only as part of a larger unit after it (i.e. interdependence of these units); and (2) there is a lack of within-group conflict such that the larger unit can be thought of as a fitness maximizing individual in its own right (i.e. there is long-term alignment of interests). Also called evolutionary social complexity.

**Phenotypic social complexity**. The extent to which a system is made from many interacting parts all coming together to contribute to a function. This is a measure of phenotypic complexity within each level of transitionary level and can be explained via multiple traits, defined as follows. **Colony size** (CS): the larger the colony, the less likely the chance of individuals to become reproductive, therefore they are selected to specialize instead. Also, there are more individuals in the first place to interact to do different jobs. From 1 to 7, the number of units within a colony for a given species, logged. **Functional sterility** (FS): this trait is important for separating termite species which have workers able to disperse to become primary reproductives (fertile workers/wood-dwelling) and those that cannot (functionally and obligately sterile workers/foraging). **Helper polyphenism** (HM): the greater the number of morphs of workers and soldiers, the greater the number of specialized groups in a colony. 1–4 morphs have been measured in this study. **Nest complexity** (NC): an extended phenotype signifying the complexity of behaviour (polyphenisms) required to create the nests around them three separate levels: (1) no structure, (2) subterranean structure, (3) above ground structure. **Obligate sterility** (OS): individuals are committed to their role as workers and therefore will be solely selected to be the most specialized and efficient worker they can be. 0 or 1 where 1 is a species which has workers unable to take over the colony or found their own and 0 is anything else.

**Soldier**. Sterile altruistic caste which are generally morphologically and behaviourally specialized for defence.

**Termite, higher**. Made up of only termite species within the family Termitidae, which only have non-flagellate gut symbionts.

**Termite, lower**. All termites other than the Termitidae. They have both flagellates and non-flagellate gut symbionts.

**True workers**. Individuals that have diverged and are part of a separate wingless line.

**Evolutionary social complexity**. The extent to which a group has become a fitness maximizing individual in its own right, with long term alignment of interest and complete interdependence.

**Wood-dwelling termites** (formally, one-piece nesters). Species where a colony will live in a single piece of wood which serves as both food and nest source. Only the winged sexuals leave the nest and when their only food source is exploited the colony will die. Species within this life type are thought to have highly flexible development and false workers which can also be described as fertile workers. These are found within the Termposidae, Kalotermitidae and some species within the Rhinotermitidae.

An alternative perspective on social complexity is a phenotypic viewpoint. Phenotypic views of social complexity focus on how social organisms look and function now, rather than on how they have evolved per se. In this sense, phenotypic social complexity can be defined as the extent to which a system is made up of many specialized and interacting parts which come together to contribute to a function (box 1) [6]. Many of the phenotypic measures of phenotypic social complexity have been derived from the study of social insects. Four readily quantifiable phenotypic measures are colony size, helper polyphenism (i.e. the degree of polymorphism among helper individuals), nest complexity and worker sterility. Colony size quantifies the number of individuals within a colony [5–14]. The larger the colony of fertile workers, the less likely the individuals are to reproduce and the more likely they are to be selected to specialize within a colony instead. Further, larger colony sizes lead to there being more individuals to interact and perform different jobs and generate complex behaviours and extended phenotypes [6–13]. Helper polyphenism captures the distinct physical variation observed among the workers and soldiers of a colony. The presence of a diverse array of worker and soldier morphs within a colony enables the formation of specialized groups that can tackle various tasks, thereby enhancing the overall functioning and productivity of the colony [6–9,14,15]. Nest complexity signifies the repertoire of behaviours colonies use to create and manage their nests (colony centres).

Evolutionary and phenotypic views of social complexity are thought to be linked by the concept of sterility due to it being an indicator of high complexity in both views. Within social insects, for instance, it is assumed that there is a positive relationship between worker sterility and all the other phenotypic social complexity traits, such that all social complexity traits can be conflated [5,16–25]. This potential relationship leads to an assumption that worker sterility can act as a proxy measure for social complexity overall. Further, it has been proposed that obligate sterility is the prerequisite of a major evolutionary transition (i.e. evolutionary social complexity). Once a species has irreversibly gained sterility, it will be selected for greater complexity in all traits in a positive feedback process [5,26]. This is due to obligate sterility removing the potential for reproductive conflict within a colony and therefore allowing for complete interdependence and alignment of interests [3–5]. Therefore, obligate sterility is seen as the key trait aligning both phenotypic and evolutionary views of social complexity.

Despite the importance of sterility, however, there are two key issues that complicate its use and interpretation as a general proxy for social complexity at large. The first is that phenotypic social complexity is likely to be a more complex and multivariate concept than previously thought. For instance, variation in traditional phenotypic measures of social complexity such as colony size, colony longevity and worker size variation, within different Hymenoptera taxa demonstrate that these measures do not always correlate with each other [6–8,27]. Furthermore, within bumblebees, some species can display high levels of phenotypic social complexity despite the presence of fertile workers [28]. The second issue is that sterility itself is complicated. For instance, some multicellular organisms can regenerate their germline (i.e. Echinoderms) and therefore have functionally sterile somatic cells, rather than obligately sterile somatic cells. This casts doubt on the importance of complete early separation of germline and soma for obligate multicellularity and, therefore, for major evolutionary transitions [19,29,30]. Multicellular species that have a functionally sterile somatic cell line, rather than an obligately sterile one, show that although there is potential reproductive conflict, it has not prevented complete interdependence and alignment of reproductive interests [1,3]. These datapoints highlight that functional sterility, rather than obligate sterility, may be of more relevance for both evolutionary and phenotypic views of social complexity and major evolutionary transitions (in both multicellularity and social insects). We require a greater understanding of atypical developmental systems if we are to fully appreciate the role of sterility in generating or maintaining social complexity [1,19].

**Figure 1.**
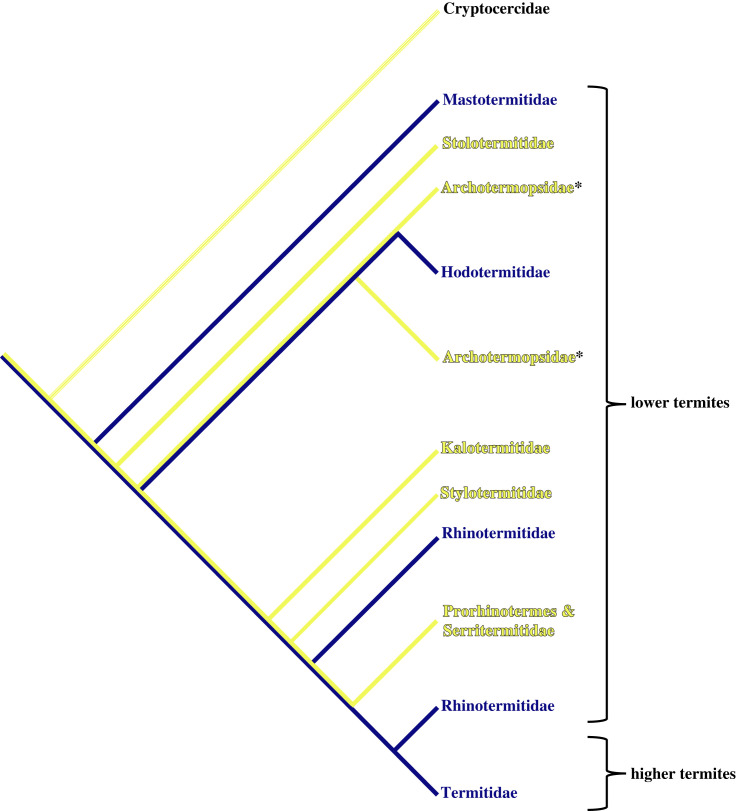
A cladogram showing the higher and lower termites as well as the major families within the termites and whether they are wood-dwelling (yellow) or foraging (blue). Cryptocercidae is the outgroup sister taxa to all termites. Taxa with asterisks are non-monophyletic.

To explore the overall importance of functional and obligate sterility in explaining phenotypic and evolutionary social complexity, we must first examine them at the highest levels of sociality within developmentally atypical systems [31,32]. The hemimetabolous termites are an extremely useful system to do this. Termites are of huge ecological and economic importance across the globe [33–36] and display a wide range of social complexities—from simple colonies to complex agricultural societies. Traditionally, they are grouped into the ‘higher’ and ‘lower’ termites, this distinction separates those species which have flagellates in their guts (lower termites) from those that do not (higher termites) (figure 1). This separation has been claimed as a defining biological difference. In terms of social complexity, however, this distinction is not particularly useful as both higher and lower termites exhibit a range of complex and simple societies [31,32,37,38]. Crucially, termites have a complex relationship with sterility—making them an ideal model taxon to investigate how this trait links to other measures of social complexity [37]. Some termites retain fully fertile workers (often called ‘wood-dwellers’ or one-piece/single-piece nesters [39]), some have functionally sterile workers which can become fertile, if necessary (‘foraging’ termites or separate piece nesters [39]), and some have fully sterile workers (also ‘foraging’ termites or separate piece nesters [40]; see figure 1) [31,32,37,40].

Here, we use trait data from the large termite collection of the Natural History Museum, London, supplementing pre-existing data, to produce the most comprehensive phenotypic social complexity trait dataset for termites to date. By doing so, we test whether obligate sterility, functional sterility or neither are able to explain variation in termite phenotypic social complexity. We make use of the dataset to answer the following questions that arise from the problems discussed above:

(1) Can either trait (functional or obligate sterility) be used a singular proxy for phenotypic social complexity in termites?
(2) Regardless of their proxy power, are there any significantly positive associations between functional and obligate sterility and the other social complexity traits?
(3) Is obligate sterility reliable as a central concept allowing phenotypic and evolutionary social complexity to be seen inseparably? Is obligate sterility necessary for higher evolutionary social complexity?

## 2. Methods

### (a) Data collection

We collected morphological trait data from termites preserved in ethanol at the Natural History Museum, London. We used head width, hind femur length and front tibia length to predict the number of worker and soldier morphs in a species (helper polyphenism). Head width is used as a proxy of body size and the two leg measures capture potential limb allometries related to foraging mode and task allocation [41]. We sampled from 300 species in total with every termite genus in the collection represented but with several genera, such as *Macrotermes*, having a greater representation to allow for their geographical spread and species richness. Where possible, 30 workers and at least 10 soldiers were sampled from each species, preferably with three individual workers and 1 soldier from 10 different colonies to prevent colony level bias. We photographed each termite specimen twice to allow for digital measurements of their morphology. To do this, we placed individual termites on blobs of K-Y jelly (Thornton & Ross) within a Petri dish to maintain their posture and covered them with ethanol to prevent them from drying out. We then used an Axio Zoom.V16 (Zeiss) to automate the photographing of each individual. We oriented individuals for a profile and a dorsal photograph. In total, we took 18 900 photographs. We used ImageJ version 1.53a to measure the head width, hind femur length and hind tibia length directly [42].

Once these measures were collected, we used the clustering analysis tool DBscan in R version 4.2.1 [43,44] to identify the number of distinct groups of workers and soldiers within each species using the morphometric measures (see electronic supplementary material, S1). This is a measure of helper polyphenism. Within the DBscan function, MinPts (the minimum number of samples seen together that can be defined as a cluster) was set at 4 so that potential human error in measuring a single individual would not cause a mistaken morph number. The functions kNNdist and kNNdisplot, from within the DBscan package, were used to calculate the k-nearest neighbour distances and plot them to identify the most appropriate eps value. The eps value dictates how close points should be to each other to be considered a part of a single cluster. To complement our estimates of helper polyphenism, we cross-referenced them with existing literature and changed the estimates where there was greater evidence for a different estimate within the literature. The script (named DBscan.md) and the relevant data can be found in the GitHub repository (see data availability section).

The acquisition of data for obligate sterility (OS), functional sterility (FS), colony size (CS) and nest complexity (NC) involved the systematic search of Web of Science for each species that was measured from the museum collections. This search was expanded to genus level when there was no data as species level. In total, 99 of the 300 species photographed could be used for these analyses as many had incomplete data relating to these traits.

Most colony size data were acquired from a single paper which has compiled the existing literature on this trait [45]. To process the data, we rounded the maximum colony size values to the nearest power of 10 and then applied the logarithm base 10 function to them. Much of the data on sterility was also previously acquired from studies compiling developmental plasticity and worker fertility data across the termites [37,46]. We define obligate sterility as a species which has workers unable to take over the colony or found their own under any circumstances (box 1). Functional sterility is defined as species which have workers able to become reproductives only under extreme circumstances, for instance where the primary reproductives have died (box 1). There are varying levels of reliability with the data, as some are long-term observational data stating that no replacement reproductive were present, some were field-based colony orphaning experiments and others laboratory-based colony orphaning [37,46]. We can only be totally certain that a species has actual obligate sterility when doing these in-depth colony orphaning experiments. These experiments help with understanding absolute developmental potential, not for understanding whether these species naturally produce replacement reproductives from workers in the field. The trait of functional sterility includes any species where the workers cannot become a primary founding reproductive, therefore including obligately sterile species. Nest complexity data were taken from the literature and defined as three separate levels: no structure, subterranean structure, above-ground structure. Species which reside in wood are making use of pre-existing structures so do not require as many building behaviours to create their nest, whereas the subterranean structures of soil-dwelling termites certainly require constructions behaviours as well as related behaviours required when creating their own nest and some defence. The creation of above-ground structures requires substantially more construction behaviours as well as defence behaviours due to potentially being more vulnerable to predators. The complete trait data matrix for the 99 species can be found in the GitHub repository (see data availability section).

### (b) Phylogeny

**Figure 2.**
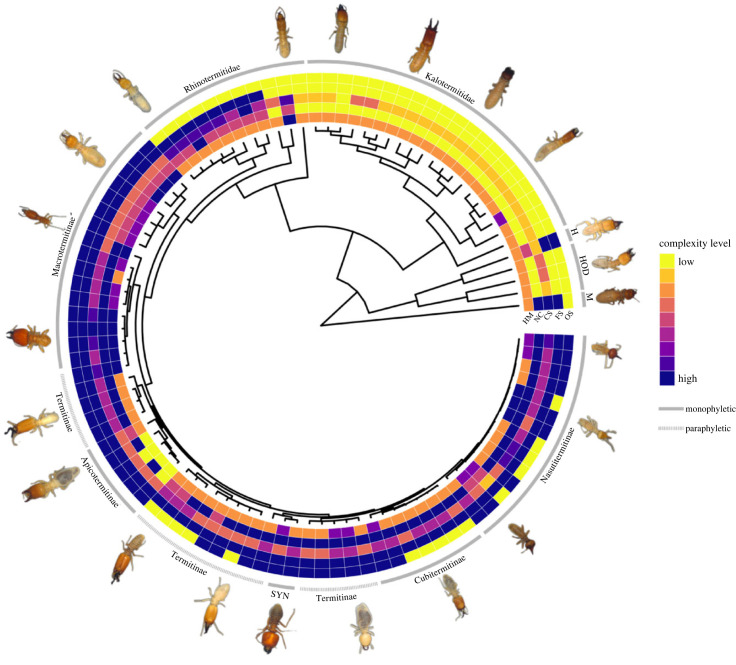
Phylogeny with the 99 species of termites used in analyses and their status in each of the five complexity traits standardized from 0 to 1 where yellow is 0 (low complexity) and blue is 1 (high complexity) in each trait: HM, helper polyphenism; NC, nest complexity; CS, colony size; FS, functional sterility; OS, obligate sterility. Abbreviated sub/family names: M, Mastotermitidae; S, Stolotermitidae; ARC, Archotermopsidae; H, Hodotermitidae; SYN, Syntermitinae. Photographs are from some of the specimens used in this study from NHM London.

We estimated a termite phylogeny from 637 termite species and nine outgroup *Cryptocercus* cockroach species as created using PyPHLAWD [47]. This is an open-source python package that creates molecular tree-building datasets from publicly available genetic data from GenBank [48], NCBI BLAST [49] and uses a Markov clustering approach [50] to infer a RAxML tree [51]. This allowed us to combine DNA from many genes for all termites that had a genus and species name in GenBank. Recent termite phylogenies informed us on the constraints needed on the tree [52–57]: Mastotermitidae, Hodotermitidae, Stolotermitidae, Kalotermitidae, Serritermitidae, Stylotermitidae and Termitidae are monophyletic families; Rhinotermitidae and Archotermopsidae are not. Subfamilies Rhinotermitinae, Apicotermitinae, Foraminitermitinae, Cubitermitinae, Syntermitinae, Macrotermitinae and Nasutitermitinae form monophyletic groups; Heterotermitinae and Termitinae do not. Following tree reconstruction, 14 taxa were trimmed that were known to be incorrectly placed, probably due to previous misidentification (figure 1). Species present in the tree but not in the data were then removed. Any remaining species missing from the tree but present in our data were manually added either to the already present genus (48 sister species added) and giving the same branch length as their sister species or when no other member of the same genus is present, we made use of pre-existing termite phylogenies to add the remainder of the species (11 newly added genera). This was done using treegraph2 [58]. Consequently, the final phylogeny we used for further analysis included 99 species [52–57]. Their branch lengths were equal to their single sister species, or an average of multiple closely related species already present in the tree (figure 2). The scripts, tree_creation.md and Figure_2_creation.md (a script to visualize the phylogeny and trait data) and the relevant data can be found in the GitHub repository (see data availability section).

### (c) Statistical analysis

All analyses used R version 4.2.1 [44]. We investigated the ability of the functional and obligate sterility traits to explain the variance in the other social complexity traits, as a test of their proxy power, by running a phylogenetic MANOVA where functional and obligate sterility (binary traits) were each in turn explanatory variables and the response variables were helper polyphenism, colony size and nest complexity (discrete traits). First, we used principal coordinate analyses (PCoAs) to summarize the discrete response data as three continuous axes of variation using the *ape* package [59]. These axes were used as response variables in a phylogenetic MANOVA which required the *phytools* and *vegan* packages [60,61]. The script (named Phylogenetic_MANOVA.md) and the relevant data can be found in the GitHub repository (see data availability section).

We also tested the associations between functional and obligate sterility, and the other social complexity traits (nest complexity, colony size and helper polyphenism). To account for non-independence due to common ancestry and the discrete nature of the data, we used a Bayesian phylogenetic mixed model approach from the package MCMCglmm version 2.34 [62]. This package uses a Markov chain Monte Carlo (MCMC) estimation approach and places the phylogenetic relationships among species as a random variable to account for the non-independence of closely related species [63]. The number of iterations, thinning and burn-in period for each pairwise comparison was by default 100 000, 50 and 5000, respectively. We used a mixed model with a threshold distribution where functional and obligate sterility are the response variables and the predictors are helper polyphenism, colony size and nest complexity. A weakly informative Gelman prior was used for fixed effects and an inverse Wishart prior for random effects, fixing the residual variance to 1 as this cannot be estimated from binary data [64–66]. We ran multiple chains and tested for convergence using the gelmon.plot function from the coda package [67]. We report the significance of a relationship using overlap of the upper and lower 95% CLs with 0. The script (named MCMCglmm.md) and the relevant data can be found in the GitHub repository (see data availability section).

## 3. Results

We found that the functional sterility trait accounts for approximately 40% of the variation in the other social complexity traits (*R*^2^ = 0.404; table 1). However, obligate sterility explains much less with only 13% variation explained in these social complexity traits (*R*^2^ = 0.131; table 1). Although neither can be seen as sufficient singular proxies for understanding termites broadly, functional sterility does explain a significant proportion of variance in the other social complexity traits. Certainly, it explains much more than obligate sterility.

**Table 1.** Phylogenetic MANOVA. Helper polymorphism, colony size and nest complexity are the response variables for both explanatory variables.

explanatory variable	d.f.	SumofSqs	*R*^2^	*F*	Pr(>f)
functional sterility	1	97.269	0.3998	65.29	0.001
obligate sterility	1	31.906	0.131	14.668	0.001

**Figure 3.**
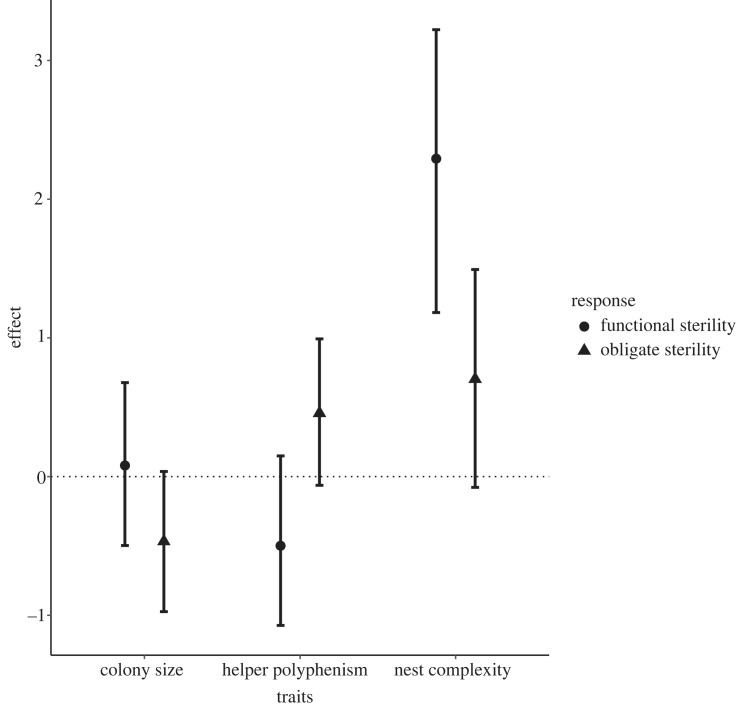
Effect plots with explanatory variables are on the *x*-axis and effect size is on the *y*-axis. The mean effect size with 95% CIs are plotted where an overlap with 0 signifies a lack of significance in the variables relationship with the response variable (circle, obligate sterility; triangle, functional sterility). This shows results from all three chains from each relationship. Summaries of results for one chain allowing for Pmcmc values are presented in table 2.

Genera that have high overall social complexity in all the traits include the Macrotermitinae and Syntermitinae (figure 2), whereas families with low overall social complexity include Kalotermitidae, Stolotermitidae and Archotermopsidae (figure 2). There are examples of species (such as *Trinervitermes bettonianus*) which have a large colony size and high polyphenism but without obligately sterile workers. We also see species such as those in the Apicotermitinae (figure 2) with small colony sizes and low polyphenism (due to their loss of the soldier caste) but workers with obligate sterility. We statistically explored the individual relationships of the different social complexity traits using MCMCglmm analysis. We found that functional sterility had a significantly positive relationship only with nest complexity, having CL 95% not overlapping 0 (l-95%, 1.114; u-95%, 3.428) (figure 3 and table 2). Obligate sterility had no significant relationship with any of the other traits (figure 3 and table 2).

**Table 2.** Summary results from a MCMCglmm chain. (*a*) Results where the response variable is functional sterility. (*b*) Results where the response variable is obligate sterility. ** outlines a pMCMC value below 0.01.

	post.mean	L-95% CI	U-95% CI	eff.samp	pMCMC
(*a*)					
(intercept)	−0.809	−4.719	2.325	1900	0.643
colony size	0.073	−0.623	0.737	1900	0.827
nest complexity	2.295	0.984	3.418	1101	0.004**
helper polyphenism	−0.505	−1.2	0.233	1900	0.173
(*b*)					
(intercept)	−1.4167	−6.6117	3.0581	2254	0.551
colony size	−0.4719	−1.0867	0.1647	1900	0.14
nest complexity	0.7011	−0.1982	1.7043	1900	0.153
helper polyphenism	0.4724	−0.1894	1.0653	1725	0.127

## 4. Discussion

### (a) Can sterility type explain termite phenotypic social complexity?

Here, we found that functional sterility explains a more significant proportion of variance in social complexity traits across termites than obligate sterility. However, it is clear that neither trait can be used as a single proxy to explain the diversity of all other social complexity traits (table 1). Instead, we argue that we should use a multivariate view of phenotypic social complexity. This is already gaining traction within the Hymenoptera literature, and clearly would be beneficial in general across the social insects and potentially across all forms of sociality [6–8,27]. The usefulness of complexity traits such as obligate sterility to represent the phenotypic social complexity of species is clearly exaggerated. It would be better to use functional sterility to explain the complexity of termites, but a more nuanced multivariate approach would capture the most variance. An overall phenotypic social complexity score for each species based upon the bringing together of the complexity traits we have explored may allow us to incorporate this greater detail. However, before this can be done in termites, traits such as colony longevity, queen-worker dimorphism and age polyethism will need to be more comprehensively collected. This will allow the truest picture possible of overall phenotypic social complexity [6–8,27]. Further exploration is needed to determine whether to assign different weights to the traits when developing this score [6–8,27]. It is likely that there will be no single approach for every question using these frameworks and data. Instead, traits may be weighted differently depending on the question of interest. But only through greater discourse are we able to decide the relative importance of each trait in evolutionary social complexity. Finally, to make our results more robust, we should try and gain more termite species trait data to reduce the problems with having only a relatively small number of independent transitions to obligate sterility.

### (b) The relationships of functional and obligate sterility with other social complexity traits

Although our data suggest that functional and obligate sterility traits cannot be used as a single proxy to explain the diversity of social complexity in termites, it could still be the case that they have significant relationships with individual traits. We found that this is only the case with nest complexity and functional sterility, however. The strong positive relationship we found between functional sterility and nest complexity is likely due, in part, to the first category of nest complexity being ‘nests which have no structure’, which is the case in all wood-dwelling species. This highlights the importance of reduced worker reproductive capacity in the evolution of more complex nesting capabilities. The lack of significance in the relationships between obligate sterility and the other phenotypic traits goes against several studies that assert that obligate sterility should cause an increase in complexity or that complexity is needed to achieve obligate sterility [5,16–19]. We cannot completely rule out the possibility that the lack of association between obligate sterility and these other traits could be due to having so few examples of obligate sterility in our present study compared with species with functional sterility or fertile workers. Further, it may well be the case that the presence of obligate sterility is still important for evolutionary social complexity by being the major prerequisite for a major evolutionary transition. However, based on the current data we have at hand, the lack of any relationship with these traits means that it has no fundamental value for quantifying phenotypic social complexity in termites.

### (c) Redefining the importance of sterility in phenotypic social complexity and therefore questioning the inseparability of phenotypic and evolutionary social complexity

The difference in importance of obligate sterility to phenotypic and evolutionary views of social complexity has already been highlighted [26]. It has been hypothesized that high phenotypic social complexity is not necessary for an increase in evolutionary social complexity (MET) to occur if obligate sterility is present [26]. This is an important and necessary step towards separating these two concepts. We clearly find that there is not a significant association between a species' level of overall phenotypic social complexity and their likelihood of having transitioned to a higher level of evolutionary social complexity, especially when this is defined by obligate sterility. Some species within the Apicotermitinae (figure 2), which have only the worker caste, small colony size and low nest complexity, have obligate sterility. It could be the case that they acquired obligate sterility while having low phenotypic social complexity. Alternatively, it could be that phenotypic complexity was high but was secondarily reduced after acquiring obligate sterility. Either way, this means there is no clear permanent positive relationship between phenotypic and evolutionary social complexity. We should instead view these as related but separate measures of complexity. By doing so, we are better able to incorporate species that do not conform to the rigid view that phenotypic and evolutionary social complexity fully align at every step. A colony that has small group size and only one morph with little to no nest structure could still have interdependence and an alignment of interests and therefore be seen as a higher individual [3].

It seems likely that each trait has its own independent selective pressures which cause the changes in their respective complexities. Further analyses exploring the relative importance of biotic and abiotic factors in selecting for these social complexity traits could provide greater understanding on the adaptive reasons for species evolving traits such as worker reproductivity or high levels of polyphenism [14].

### (d) Inclusivity of atypical organisms sheds light on evolutionary social complexity

We must be more inclusive to developmentally atypical organisms when outlining the prerequisites for a major evolutionary transition if we are to create a generalized framework for all life [1,19]. This includes systems that seemingly harbour potential conflict, but which have found ways to prevent this from becoming actual conflict over evolutionary time while still allowing for increases in evolutionary social complexity [1,19]. Within multicellular groups, the presence of units able to become germline or soma at any point in the group's life traditionally would prevent them from being defined at a higher level of individuality [19]. However, it has been shown in some metazoan lineages that species which clearly have interdependence and aligned interests also do not always have complete segregation of germline and soma, i.e. have early separation of a germline [19]. This is also the case in termites, which often have workers within highly complex colonies able to reproduce under extreme circumstances. We have shown in this study that obligate sterility is not an adequate singular proxy for phenotypic social complexity, nor does it correlate with any of the present social complexity traits within termites. We have outlined the need for decoupling phenotypic social complexity from evolutionary social complexity as a consequence. However, it may be necessary to go a step further; it may also be the case that obligate sterility is unnecessary to allow complete interdependence and long-term alignment of interests within the group. The closed nature of these systems with functional sterility (note that some wood-dwelling termites with fertile workers exhibit colony fusion [68]), whereby the replacement reproduction by a worker is only occurring under extreme circumstances like their parents dying, means there is less potential conflict compared with replacement worker reproduction seen in Hymenoptera [4]. Consequently, the evolutionary retention of worker reproductivity could be a group level adaptation to variable environments, present in many atypical systems like the termites and basal metazoans.

Furthermore, the alternative strategies to interdependence and alignment of interests which are not reliant on obligate sterility, shown in developmentally modular organisms like plants, should also be incorporated into our frameworks [19,29,30]. It may be the case that the highly modular but extremely complex siphonophores, Hydrozoans within the phylum Cnideria, are another novel example of a higher individual [4,69,70]. They appear to have complete interdependence and aligned interests of multicellular replicated units (zooids) coming together for the higher individual. The more inclusive we are to these developmentally atypical organisms, the more inclusive we become to alternative strategies which can produce a higher individual. In the future, we must include as many clades as possible to understand the true spectrum of individuality [1]. Wood-dwelling termites have been shown to generally have the lowest levels of social complexity in all the traits we have explored in this study, but this is only compared with other termite species. Comparisons that include the full spectrum of sociality within the Blattodea may shed more light on this discussion.

## 5. Conclusion

Creating a framework where we can compare the phenotypic and evolutionary processes by which the complexity of life on earth has evolved is invaluable. However, such a unifying concept cannot be explained so simply if we are to include the diversity of all life. Here, we have shown that the diversity of phenotypic social complexity traits such as colony size, nest complexity and worker polyphenism cannot be explained fully by functional and obligate sterility traits. Therefore, instead of a singular proxy for phenotypic social complexity, we must use a multivariate approach to explain its true diversity within termites and more broadly across all sociality if we are to step closer towards this unifying concept. Furthermore, we find that there is a lack of significant association between either functional or obligate sterility with the other social complexity traits, so we should not conflate these traits. Consequently, we outline that phenotypic and evolutionary social complexity (based on individuality) are not necessarily fully in line but instead should be seen as distinct but interacting frameworks if we are to fully understand what is required to transition to higher individuality. By turning our sights to the developmentally atypical termites, we broaden this understanding, which allows us to find greater and more accurate parallels across major evolutionary transitions, such as that between termites and siphonophores as superorganisms and plants, and some metazoan lineages as multicellular organisms.

## Data Availability

The scripts and additional data for this manuscript can be found in the Dryad repository [71]. Data and scripts to create figures and to repeat the analyses outlined in this study can be found in the GitHub repository: https://github.com/lewisrevely/Diversity-of-Social-Complexity-in-Termites.git Supplementary material is available online [72].
